# The First Known Case Report of a Novel Homozygous Nonsense Variant in the OSBPL9 Gene Associated With Fetal Cerebral Ventriculomegaly, Cerebellar Hypoplasia, and Arthrogryposis Multiplex

**DOI:** 10.7759/cureus.80010

**Published:** 2025-03-04

**Authors:** Parag M Tamhankar, Tushar Kachhadiya, Vasundhara Tamhankar, Pramila G Menon, Shalin Vaniawala, Shilpa M Mithbawkar

**Affiliations:** 1 Pediatrics, Dr. D.Y. Patil Medical College, Hospital and Research Centre, Dr. D.Y. Patil Vidyapeeth (Deemed to be University), Pune, IND; 2 Genetics, Centre for Medical Genetics, Mumbai, IND; 3 Genetics, SN Gene Lab Pvt Ltd, Surat, IND

**Keywords:** arthrogryposis multiplex congenita, cerebellar hypoplasia, fetal ventriculomegaly, novel mutation, osbpl9, snp microarray, whole-exome sequencing

## Abstract

Oxysterol-binding protein-like protein 9 (*OSBPL9*) is a member of a large eukaryotic gene lipid transport protein family that mediates the exchange of sterols and phospholipids between the trans-Golgi network and the endoplasmic reticulum. Denovo missense mutations in the *OSBPL9* gene have been previously reported to be associated with intellectual disability. Herein, we report for the first time, to the best of our knowledge, a novel homozygous nonsense variant in the *OSBPL9* gene in a consanguineous family with two fetuses with cerebral ventriculomegaly, cerebellar hypoplasia, and arthrogryposis multiplex. Whole exome sequencing and homozygosity mapping by chromosomal microarray identified one fetus to be homozygous for a novel nonsense variant chr1-51760720C>CAAT or c.615_616insTAA or p.Pro206*. Exome sequencing identified the asymptomatic parents as carriers for the same variant, indicating an autosomal recessive inheritance pattern.

A review of medical literature using databases such as PubMed/MEDLINE (Medical Literature Analysis and Retrieval System Online), and Google Scholar did not reveal any case with the *OSBPL9 *variant and fetal malformations such as ventriculomegaly, cerebellar hypoplasia, and arthrogryposis multiplex. A protein network analysis using the STRING (Search Tool for Retrieval of Interacting Genes/Proteins) database showed close interactions between the *OSPBPL9, OSBP, PI4K2A, PIP5K1C, PI4KA, CERT1, EXOSC3, RARS2, VRK1, *and *TSEN54* genes but no interactions with the *L1CAM, KIDINS220,* and *KIAA1109* genes. These proteins are important for the metabolism of sphingomyelin, sterol, and lipids such as phosphatidylinositol and ceramide in the cell. Mutations in these proteins are known to cause related genetic disorders, which include structural brain abnormalities, fetal arthrogryposis, and intellectual disability as a phenotype.

This is the first known report of a homozygous variant in the *OSBPL9* gene in a recessive inheritance pattern, and the first report of association with a fetal anomaly phenotype. Previously, only two cases with an *OSBPL9* gene variant have been documented in the literature, showing a sporadic autosomal dominant inheritance pattern. Thus, this case report expands the phenotype of *OSBPL9* gene-related human disease. This case report will aid clinical diagnosis, genetic counseling, and preventive strategies such as prenatal diagnosis and/or preimplantation genetic diagnosis in families affected with *OSBPL9* gene variants. The limitation of this study is the lack of RNA, protein, cellular, or animal model studies or functional studies to confirm this association.

## Introduction

Fetal cerebral ventriculomegaly is diagnosed when the lateral ventricle size is more than 10 mm on antenatal ultrasound. It is classified as mild when the lateral ventricle size is between 10 and 15 mm and severe when more than 15 mm. The causes of ventriculomegaly can be chromosomal disorders, single-gene disorders, or fetal congenital infections. Non-isolated ventriculomegaly includes those entities with additional anomalies such as cerebral/cerebellar hypoplasia/malformations, congenital heart defects, intrauterine growth retardation, etc. Recent studies have revealed that up to 45% of severe ventriculomegaly can have genetic etiologies diagnosed on chromosomal microarray and exome sequencing [1]. Fetal arthrogryposis multiplex (arthron = joint) is a condition that consists of contractures affecting two or more joints prenatally. Incidence is 1 in 3000 live births; however, the antenatal incidence may be higher implying high mortality. This syndrome is also known as fetal akinesia deformation sequence. Additional features include hydrops, intrauterine growth retardation, facial anomaly, skin pterygia, hypoplastic palmar dermal ridges, hypoplastic lungs, and polyhydramnios. Etiology can be primary due to mutations in chromosomes or genes or secondary due to extrinsic etiology leading to in-utero crowding such as oligohydramnios, uterine abnormalities, and twin pregnancies. Chromosomal disorders include trisomy 18 or trisomy 8. Single-gene etiologies consist of mutations in one of the known 83 genes [2,3]. Some of the important causative genes for fetal arthrogryposis multiplex are *ACTA1, NEB, BIN1, FLNB, LAMA2, CHRNA1, CHRND, CHRNG, PIP5K1C, POMGNT1, POMT1, COL6A3, ZMPSTE24, LMNA, ANTXR2, DMPK, MTM1, MTMR1,* and others [3]. A combination of fetal ventriculomegaly, cerebellar hypoplasia, and arthrogryposis has been reported in association with mutations in genes such as *KIDINS220, KIAA1109, L1CAM, PI4KA, PI4K2A, TSEN54, EXOSC3, RARS2, VRK1,* and *TSEN54* (genes related to neurological development rather than musculoskeletal development) [4-8]. Herein, we report a consanguineous family with fetal cerebral ventriculomegaly, cerebellar hypoplasia, and arthrogryposis multiplex associated with a homozygous novel nonsense mutation in the *OSBPL9* gene. This is the first known report of a homozygous variant in the *OSBPL9* gene in a recessive inheritance pattern and the first report of association with a fetal anomaly phenotype, to the best of our knowledge.

## Case presentation

**Figure 1:**
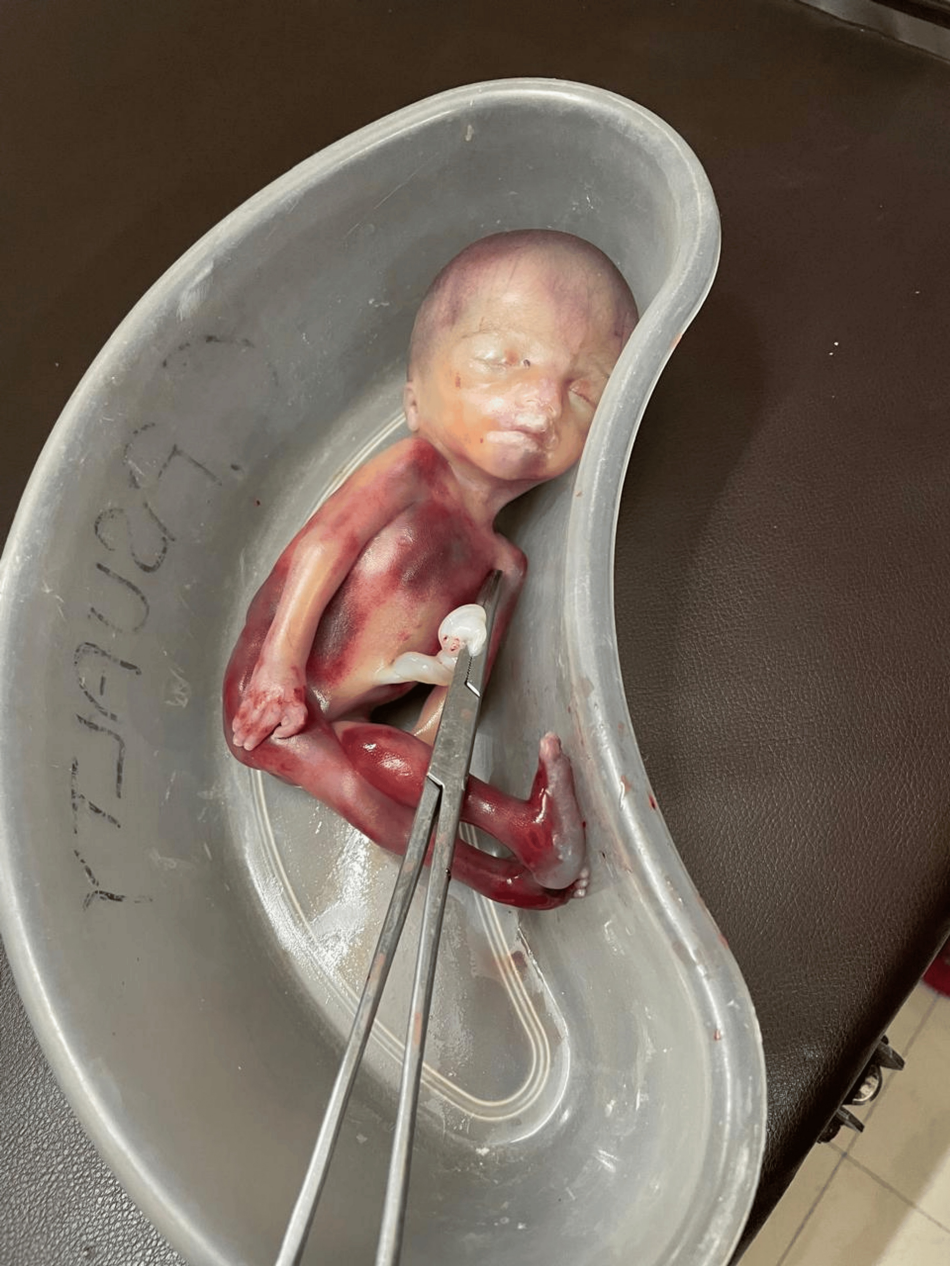
Photographs of the first fetus taken postnatally Photographs of the first fetus taken after delivery by the referring physician revealed a female fetus with a large head, ocular hypertelorism, long philtrum of the lip, downturned lower lip, arthrogryposis multiplex in the form of hyperextended elbows, flexed hips, and hyperextended knees bilaterally. Written and informed consent has been taken from the patient's family to show the uncovered face of the fetus and can be provided on request. This is essential to appreciate the facial dysmorphism in the fetus.

A couple married in a third-degree consanguineous union presented with a history of first pregnancy, a natural conception, affected with severe cerebral ventriculomegaly, cerebellar hypoplasia, and arthrogryposis multiplex detected by antenatal ultrasound at 21 weeks of gestation. They discontinued the pregnancy as per medical advice that the fetus is likely to have adverse neurodevelopmental outcomes. Detailed ultrasound reports were not available. Photographs of the fetus taken postnatally by the referring physician revealed a female fetus with a large head, ocular hypertelorism, long philtrum of the lip, downturned lower lip, arthrogryposis multiplex in the form of hyperextended elbows, flexed hips, and hyperextended knees bilaterally (Figure 1).

**Figure 2:**
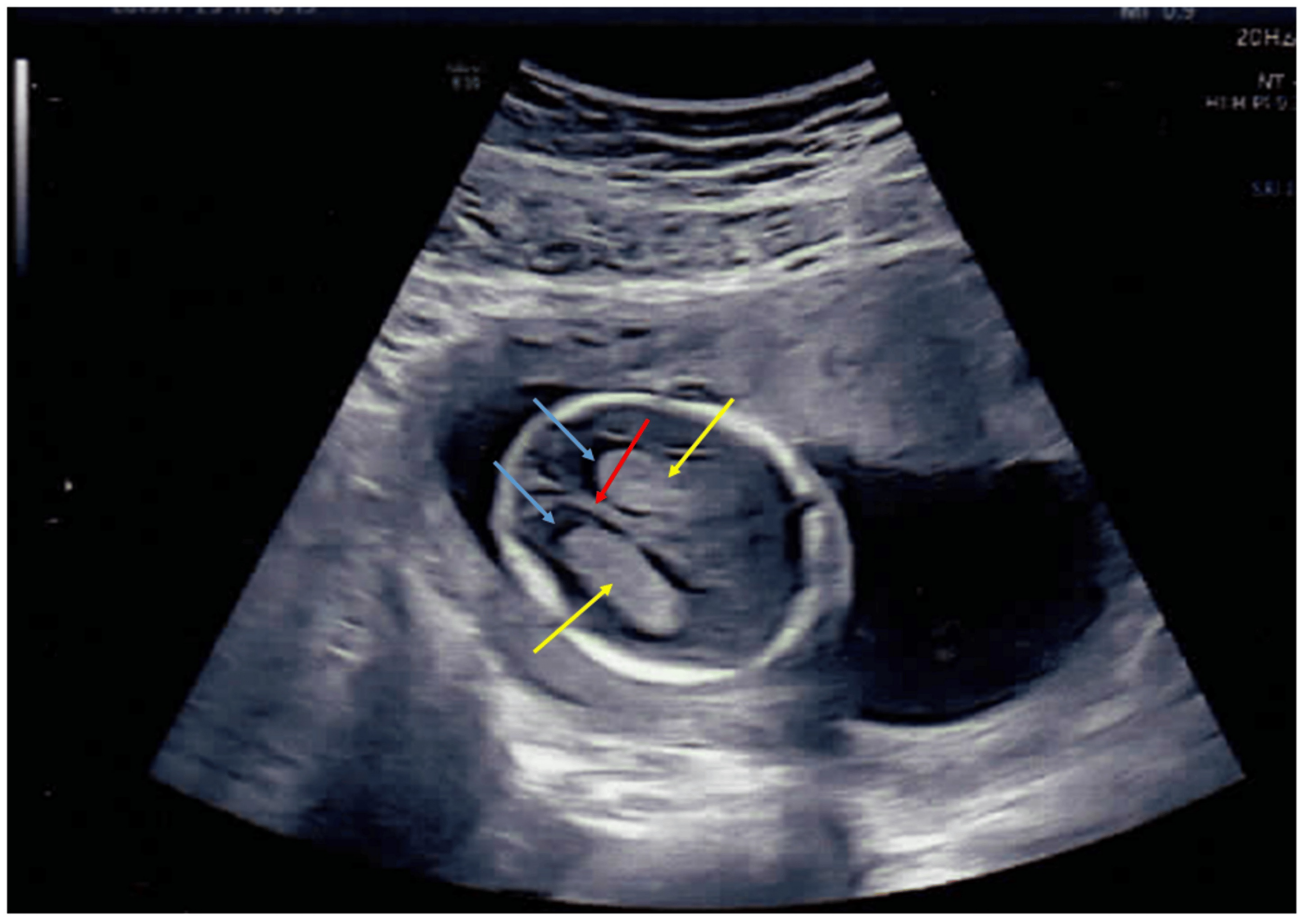
Antenatal ultrasound of the fetal skull around 15 weeks 3 days gestation in the couple's second pregnancy Antenatal ultrasound showing a transverse view of the the fetal skull at the level of the lateral ventricles showing normal intracranial anatomy around 15 weeks 3 days of gestation The choroid plexus (yellow arrows), lateral ventricles (blue arrows) and the midline falx cerebri are seen (red arrow).

**Figure 3:**
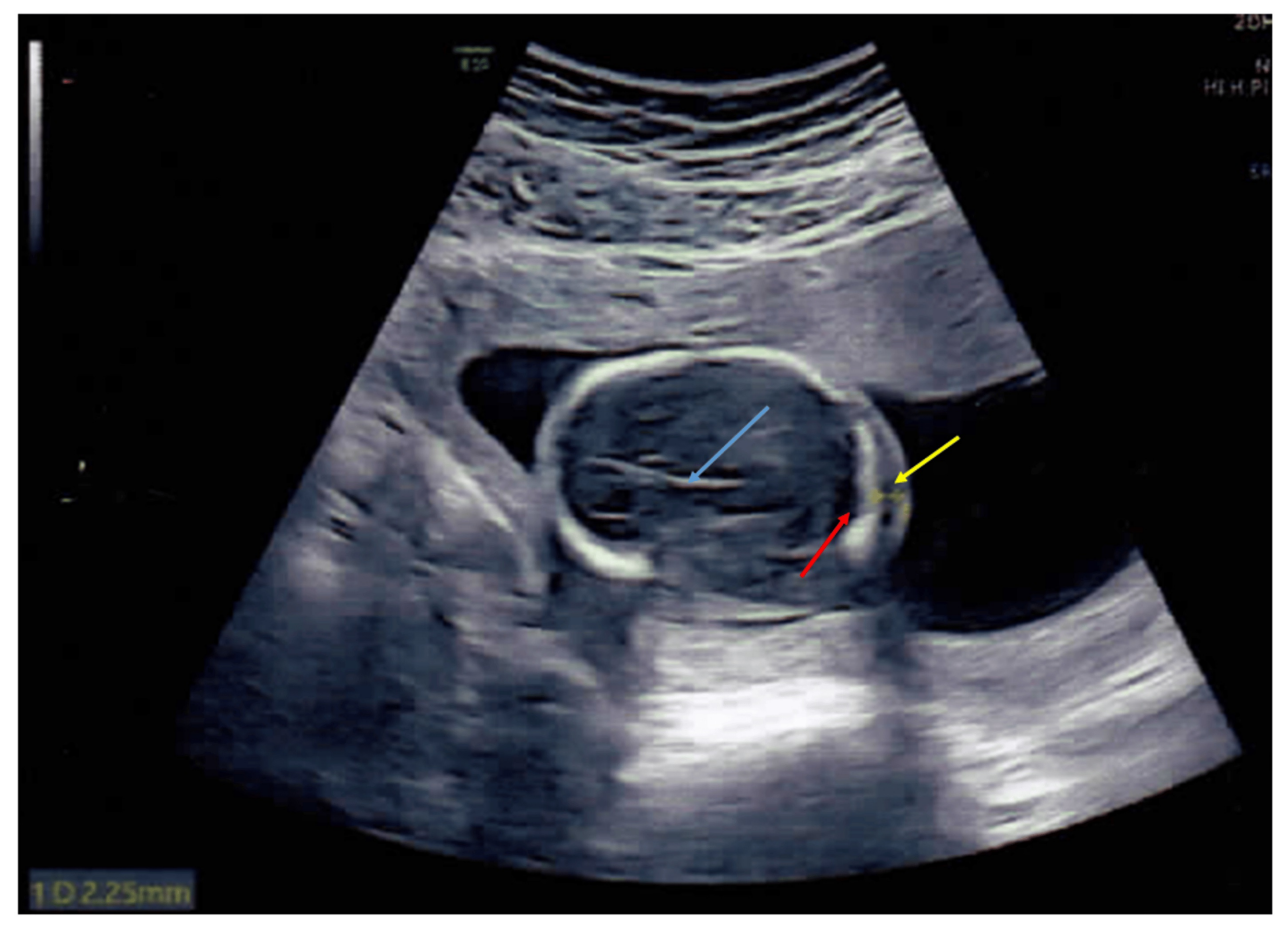
Antenatal ultrasound of the fetal skull around 15 weeks 3 days gestation in the couple's second pregnancy Antenatal ultrasound of the fetal skull around 15 weeks 3 days of gestation with a transverse view at the level of the cisterna magna (normal) (red arrow) and a normal nuchal fold (yellow arrow) of 2.25 mm is seen. A midline falx cerebri is seen (blue arrow).

**Figure 4:**
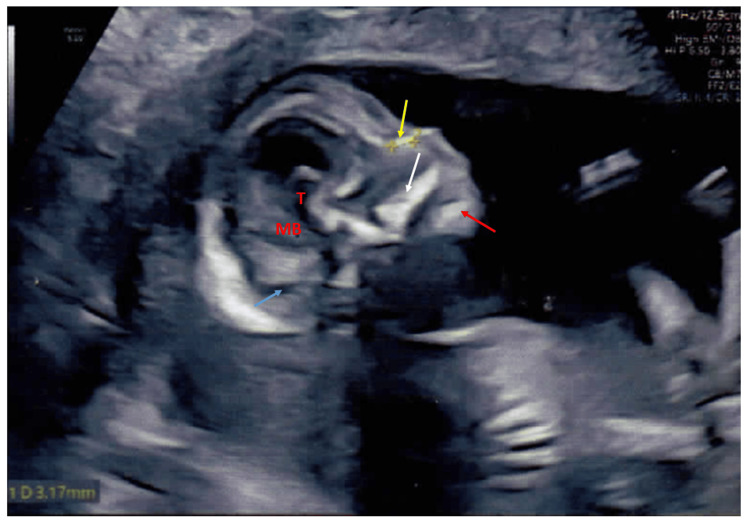
Antenatal ultrasound in the couple's second pregnancy around 15 weeks 3 days of gestation Antenatal ultrasound in the couple's second pregnancy around 15 weeks 3 days of gestation with a sagittal view of the skull with normal intracranial anatomy. Normal nasal bone (yellow arrow) measuring 3.17 mm, maxillary bone (white arrow), mandible (red arrow), normal thalamus (T), midbrain (MB), and normal cisterna magna (blue arrow) are seen.

**Figure 5:**
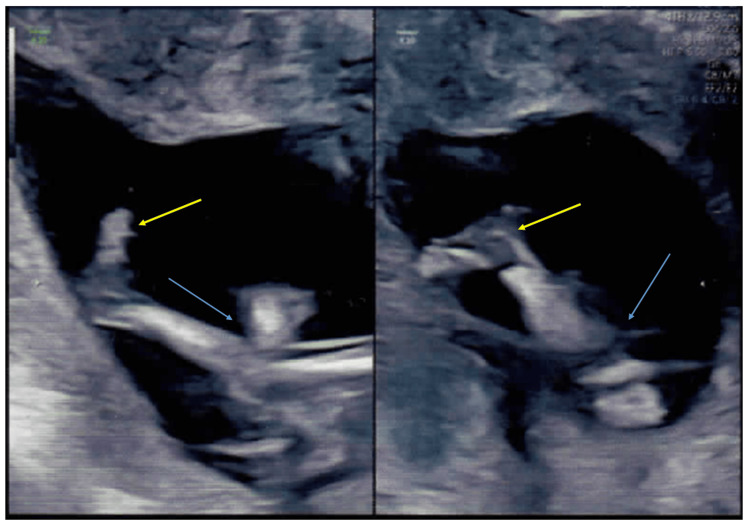
Antenatal ultrasound of the fetus in the second pregnancy around 15 weeks 3 days gestation showing hyperextended knees Antenatal ultrasound of the fetus in the second pregnancy around 15 weeks 3 days showing early signs of arthrogryposis. Both knees are hyperextended (blue arrows) and feet (yellow arrows) are normal.

No genetic tests were conducted on the first fetus. The couple was followed up during their second pregnancy, also a natural conception. The ultrasound performed at 15 weeks 3 days revealed normal anthropometry but reduced fetal movements and hyperextended knees (Figures 2-5).

**Figure 6:**
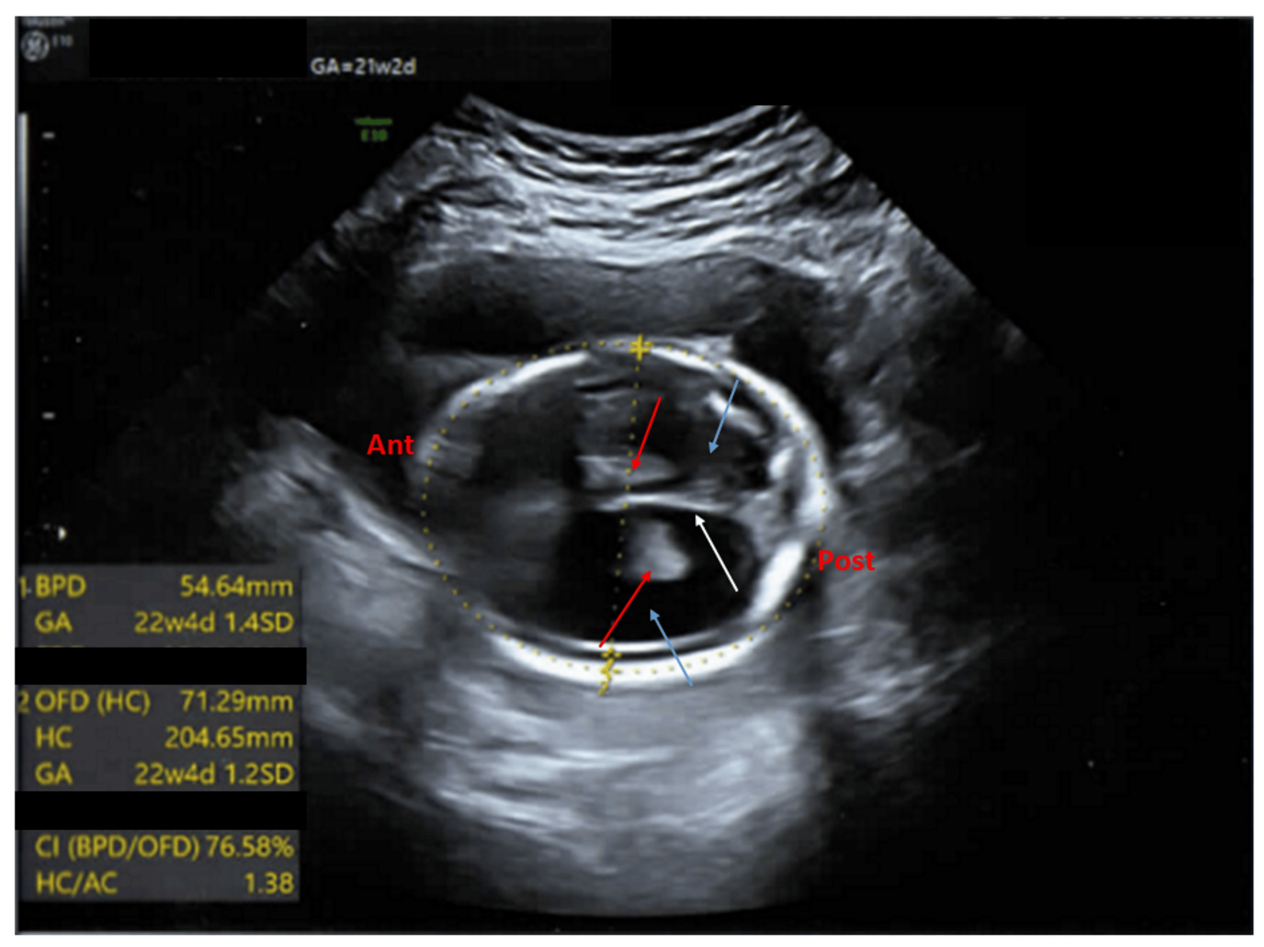
Antenatal ultrasound of the fetus in the second pregnancy showing cerebral ventriculomegaly around 21 weeks 2 days gestation Antenatal ultrasound of the fetus in the second pregnancy showing a transverse view of the fetal skull (ant: anterior side, post: posterior side) around 21 weeks 2 days (from the last menstrual period) showing dilated cerebral ventricles (blue arrow), dangling choroid plexus (red arrows), and normal falx cerebri (white arrow). The biparietal diameter (BPD) and head circumference (HC) are increased (corresponding to 22 weeks 4 days gestational age (GA).

The fetal biparietal diameter was 30.31 mm (mean for 15 weeks 3 days), head circumference was 114.52 mm (mean for 15 weeks 4 days), abdominal circumference was 92.5 mm (mean for 15 weeks 3 days), femur length was 16.88 mm (mean for 15 weeks). The intracranial anatomy was normal. The amniotic fluid index was 14. The follow-up ultrasound performed at 21 weeks revealed severe cerebral ventriculomegaly, cerebellar hypoplasia, and arthrogryposis multiplex. The fetal biparietal diameter was 54.64 mm (increased) (mean for 22 weeks 4 days) and the head circumference was 204.65 mm (increased) (mean for 22 weeks 4 days) (Figure 6).

**Figure 7:**
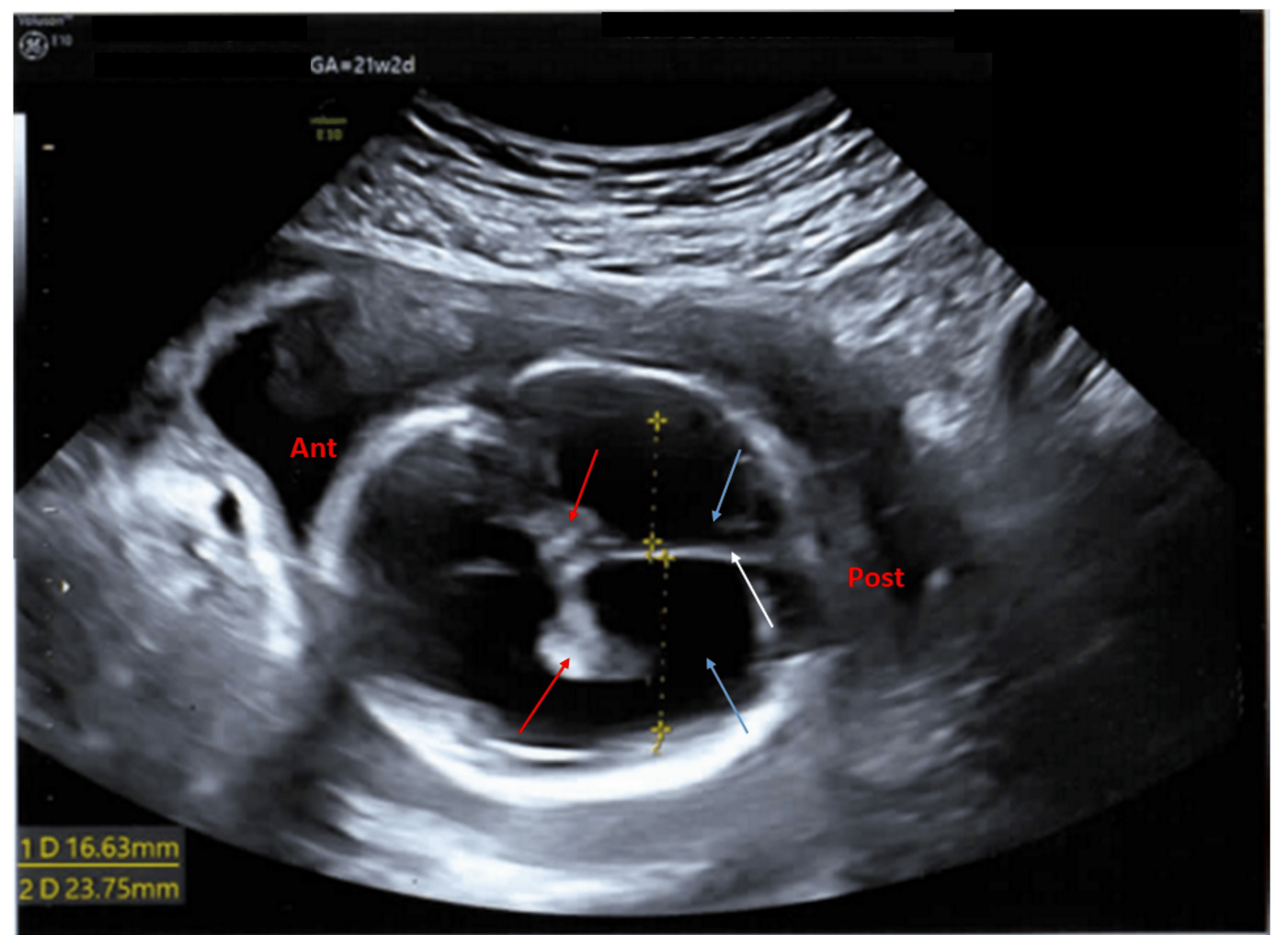
Antenatal ultrasound of the fetus in the second pregnancy showing cerebral ventriculomegaly Antenatal ultrasound of the fetus shows a transverse view of the fetal skull (ant: anterior side, post: posterior side) around 21 weeks 2 days gestation, with dilated cerebral ventricles (blue arrows) (measuring 16.63 mm and 23.75 mm in diameter) showing a dangling choroid plexus (red arrows); the falx cerebri is normal (white arrow).

The abdominal circumference was 148.07 mm (mean for 20 weeks 1 day), femur length was 33.41 mm (mean for 20 weeks 3 days), the right cerebral lateral ventricle was 16.63 mm (severely increased), the left cerebral lateral ventricle was 23.5 mm (severely increased), and the choroid plexuses were dangling (Figure 7).

**Figure 8:**
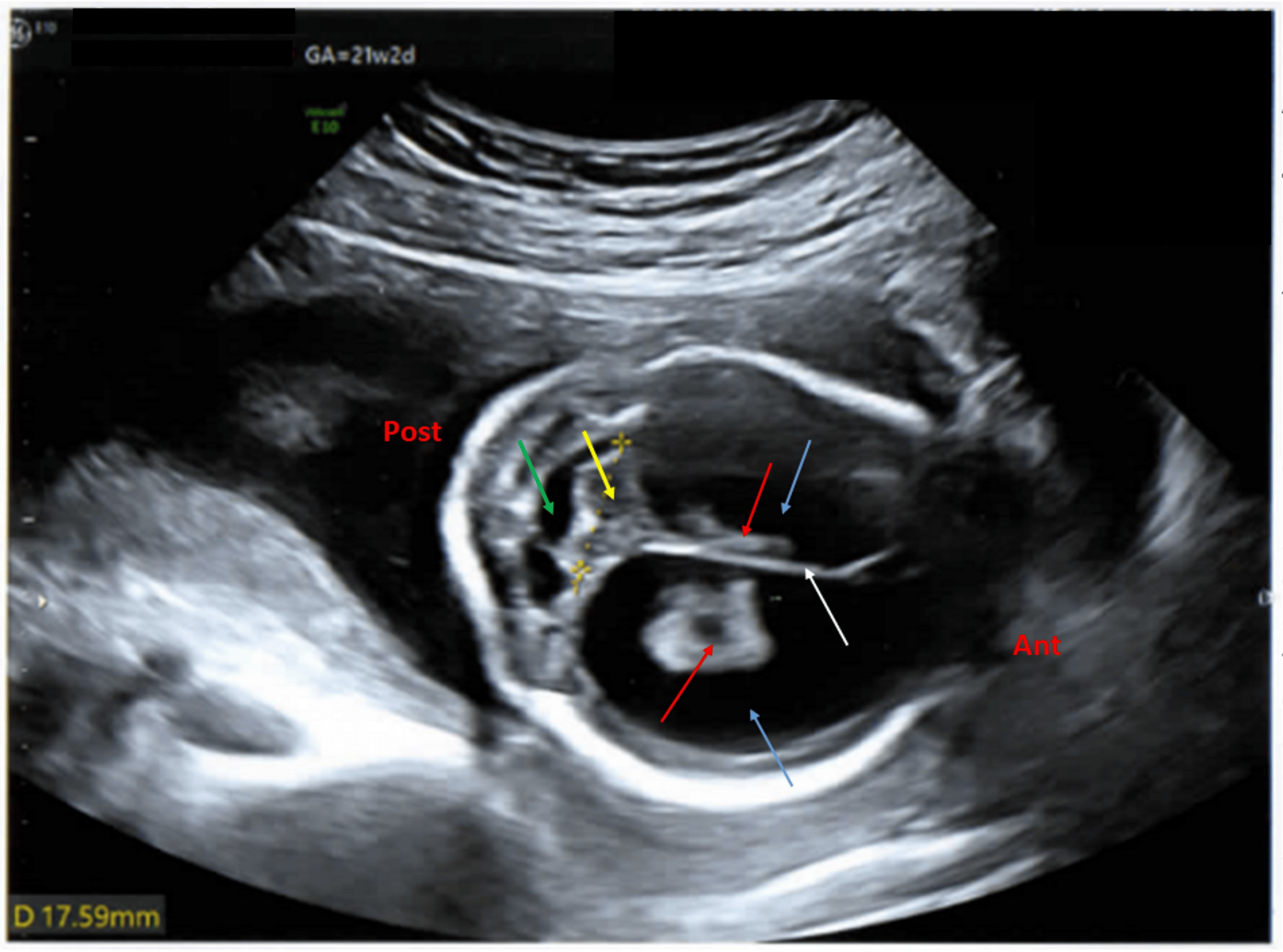
Antenatal ultrasound of the fetus in the second pregnancy at around 21 weeks 2 days gestation showing cerebral ventriculomegaly and cerebellar hypoplasia Antenatal ultrasound of the fetus in the second pregnancy showed a transverse view of the fetal skull at the level of the posterior fossa (ant: anterior, post: posterior), around 21 weeks 2 days gestation, showing dilated cerebral ventricles (blue arrows), cerebellar hypoplasia (yellow arrow), the trans-cerebellar diameter was 17.59 mm (6 percentile, z score is minus 1.59), cisterna magna was normal (green arrow), and falx cerebri was normal (white arrow).

The trans-cerebellar diameter was 17.59 mm (6 percentile, z score is minus 1.59) (Figure 8).

**Figure 9:**
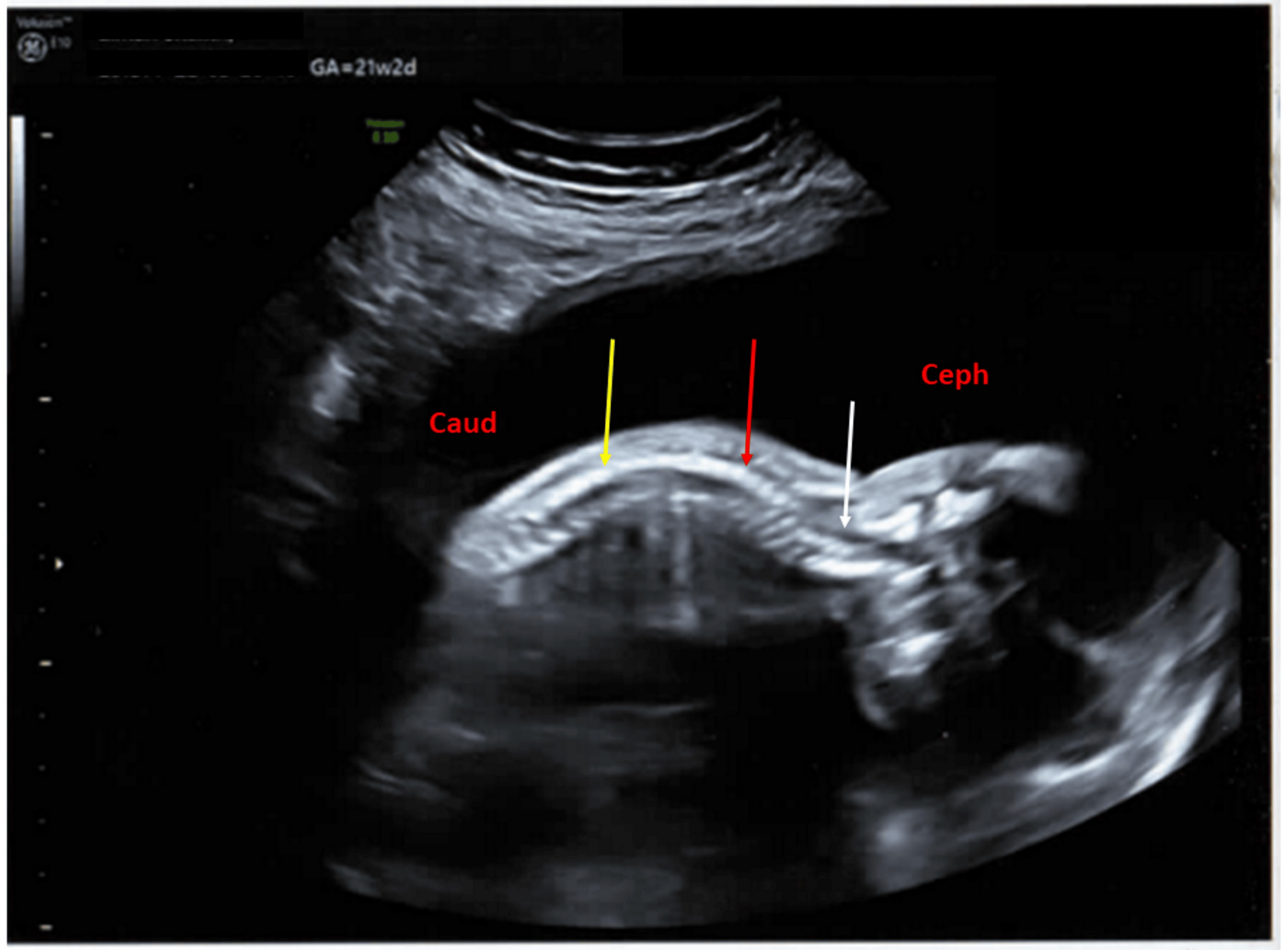
Antenatal ultrasound of the fetus in the second pregnancy showing the fetal spine Antenatal ultrasound of the fetus in the second pregnancy at around 21 weeks 2 days gestation, showing a sagittal view of the spine (ceph: cephalic side, caud: caudal side) with normal anatomy; the cervical (white arrow), thoracic (red arrow), and lumbar (yellow arrow) segments are seen with normal curvature.

The fetal spine was normal (Figure 9).

**Figure 10:**
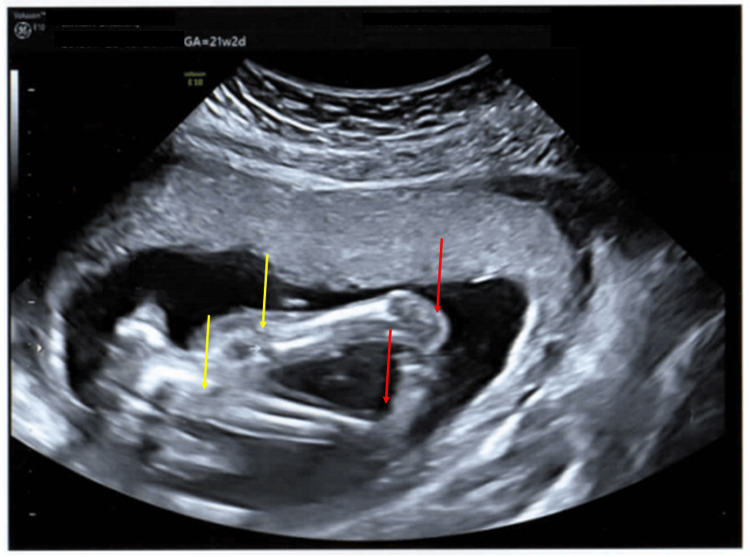
Antenatal ultrasound of the fetus in the second pregnancy at around 21 weeks 2 days gestation showing arthrogryposis Antenatal ultrasound of the fetus in the second pregnancy at around 21 weeks 2 days gestation showing signs of arthrogryposis in the form of hyperextended knees (yellow arrows) and club feet (red arrows).

The fetal movements were reduced, knees were hyperextended, and bilateral club feet were present (Figure 10).

**Figure 11:**
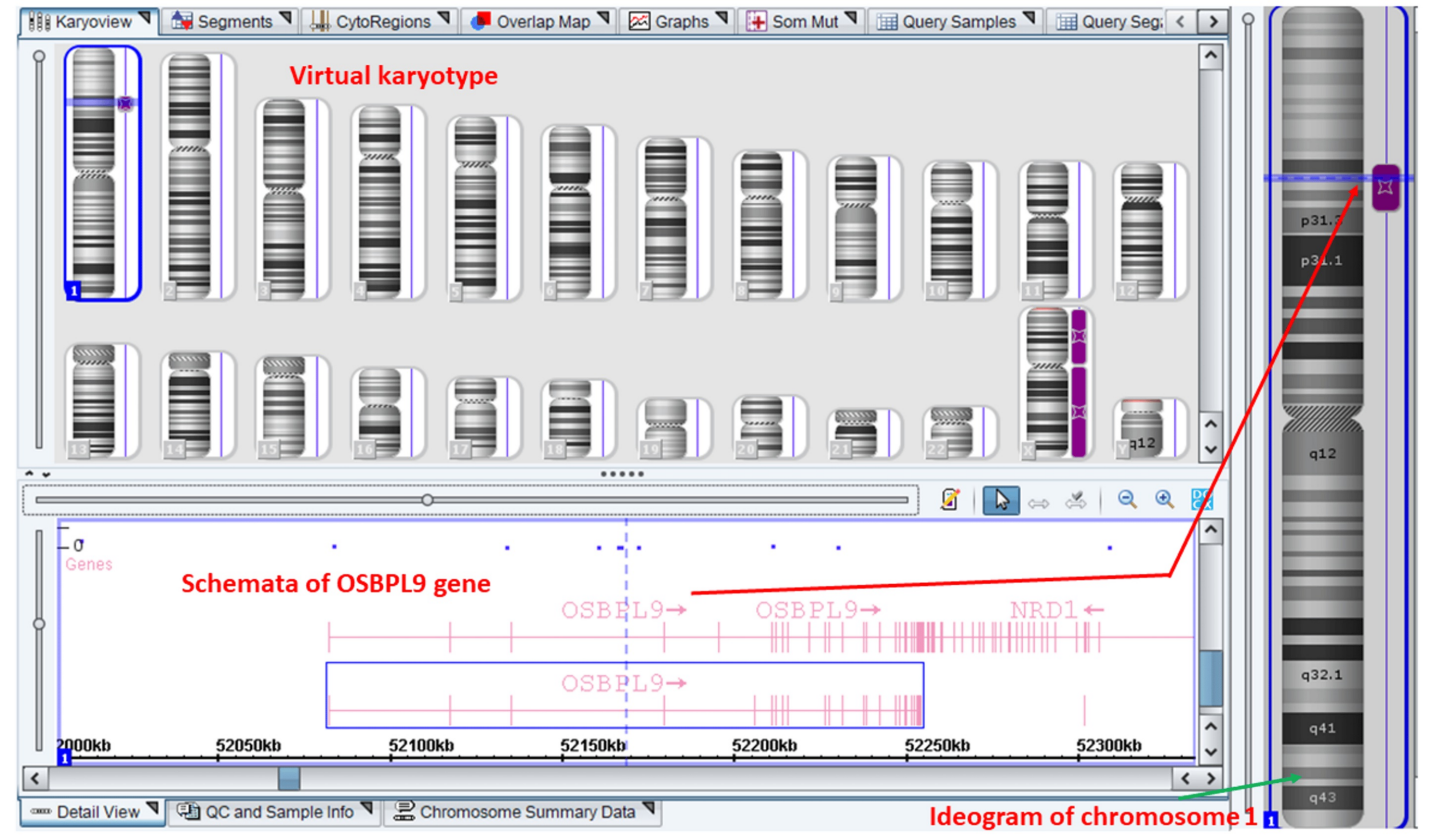
Chromosomal microarray of the fetus in the second pregnancy Virtual karyogram of fetal chromosomal microarray on CytoScan™ (Thermo Fisher Scientific Inc., Waltham, Massachusetts, US) 750K Chip shows no significant chromosomal deletion or duplication but there is a region of loss of heterozygosity (LOH) depicted by purple bands next to chromosome ideograms. LOH at the chromosome 1p arm includes the *OSBPL9* gene (red arrow). The fetus was male, hence, the X chromosome LOH is shown. LOH: loss of heterozygosity

The long bones were normal in length. The umbilical cord showed three vessels. The heart, cardiac outflow tracts, and lungs were normal. The abdominal and thoracic situs appeared normal. The kidneys, bladder, stomach, and bowel pattern were normal. The amniotic fluid index was 16. Amniocentesis was done for fetal DNA diagnosis. Chromosomal microarray performed on Applied Biosystems™ CytoScan™ Optima 750K chip (Thermo Fisher Scientific Inc., Waltham, Massachusetts, US) did not reveal any significant aneuploidies or microdeletion-duplication syndromes. Significant locus of loss of heterozygosity (LOH) of size 13,759 kilobases was present at the following site: arr[GRCh37] 1p33p31.3(48,288,540-62,047,663)hmz (long arm of chromosome 1). The *OSBPL9* gene (chromosome locus 9q34.13) is present among 90 genes in the 13.7 megabases region of LOH on chromosome 1 (Figure 11).

**Figure 12:**
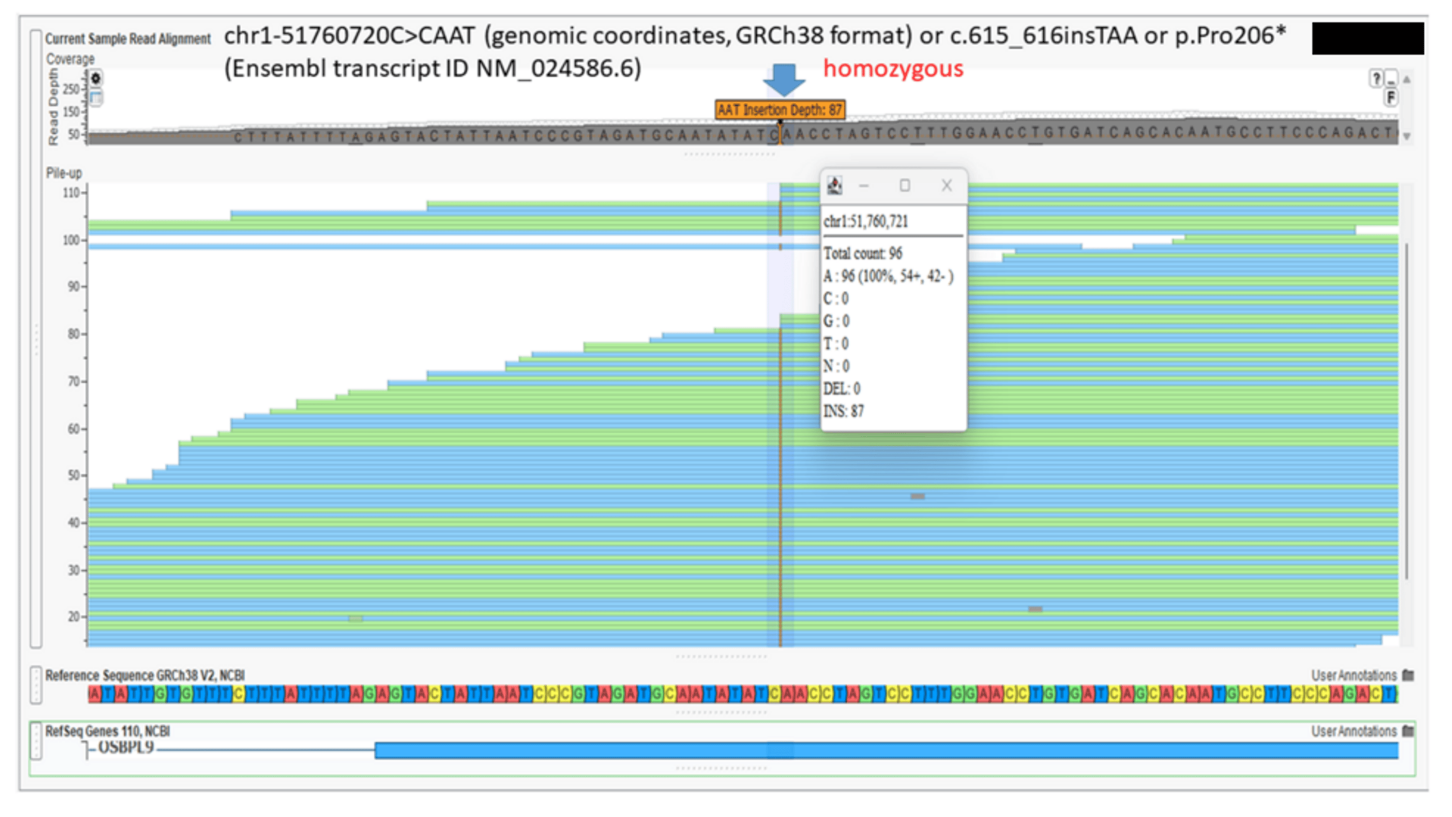
Homozygous nonsense variant in the OSBPL9 gene identified in the second fetus Screenshot of the Integrated Genomic Viewer (VarSeq; Golden Helix Inc., Bozeman, Montana, United States) of the binary alignment map (BAM) file of the whole exome sequence data for the affected fetus shows homozygous variant in the *OSBPL9* gene chr1-51760720C>CAAT (genomic coordinates, GRCh38 format) or c.615_616insTAA or p.Pro206* (Ensembl transcript ID NM_024586.6). The position of the variant is shown by the blue arrow.

A whole exome analysis (20321 genes with 37 mitochondrial genes) (Twist exome panel 2.0 with additional mitochondrial gene coverage added; Twist Biosciences Inc., South San Francisco, California, US) was performed for the fetus. In addition, to single nucleotide variants and small insertion-deletions, copy number variants were detected from the targeted sequence data using ExomeDepth (version 1.1.10) and this bioinformatics pipeline has been clinically validated [9]. Polymerase chain reaction (PCR) performed for toxoplasma, rubella virus, and cytomegalovirus was negative. This lab has been accredited by the National Accreditation Board for Testing and Calibrating Laboratories (NABL) of India. The fetus was identified to be homozygous for a novel nonsense variant (premature termination codon) chr1-51760720C>CAAT (genomic coordinates, GRCh38 format) or c.615_616insTAA or p.Pro206* (Ensembl transcript ID NM_024586.6) (read depth: 96x) (located in exon 10 out of 24 coding exons) (Figure 12).

**Figure 13:**
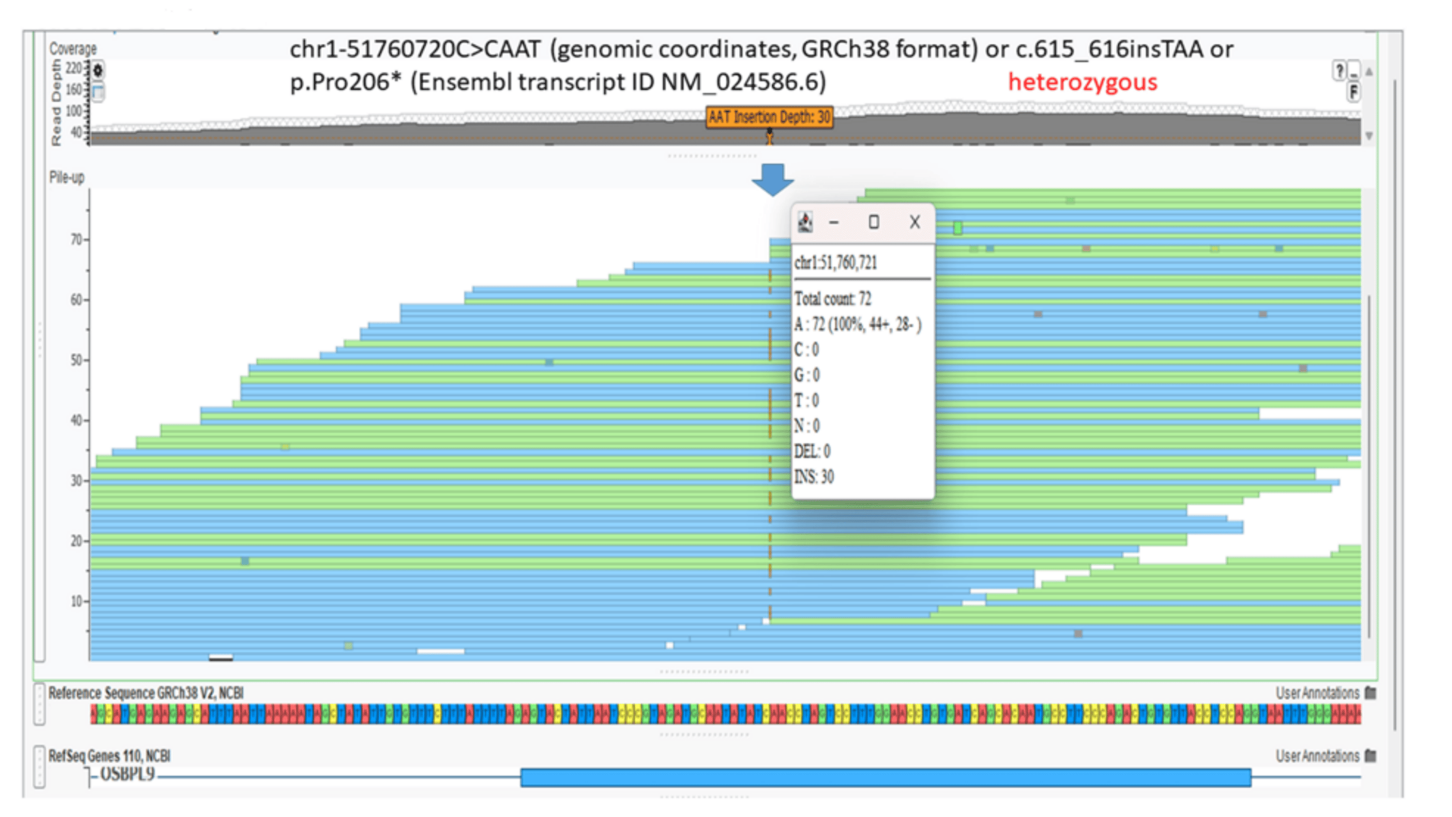
Heterozygous variant in the OSBPL9 gene identified in the mother on whole exome sequencing Screenshot of the Integrated Genomic Viewer (VarSeq; Golden Helix Inc., Bozeman, Montana, United States) of the binary alignment map (BAM) file of the whole exome sequence data for the carrier mother shows heterozygous mutation in the *OSBPL9* gene chr1-51760720C>CAAT (genomic coordinates, GRCh38 format) or c.615_616insTAA or p.Pro206* (Ensembl transcript ID NM_024586.6). The position of the variant is shown by the blue arrow.

**Figure 14:**
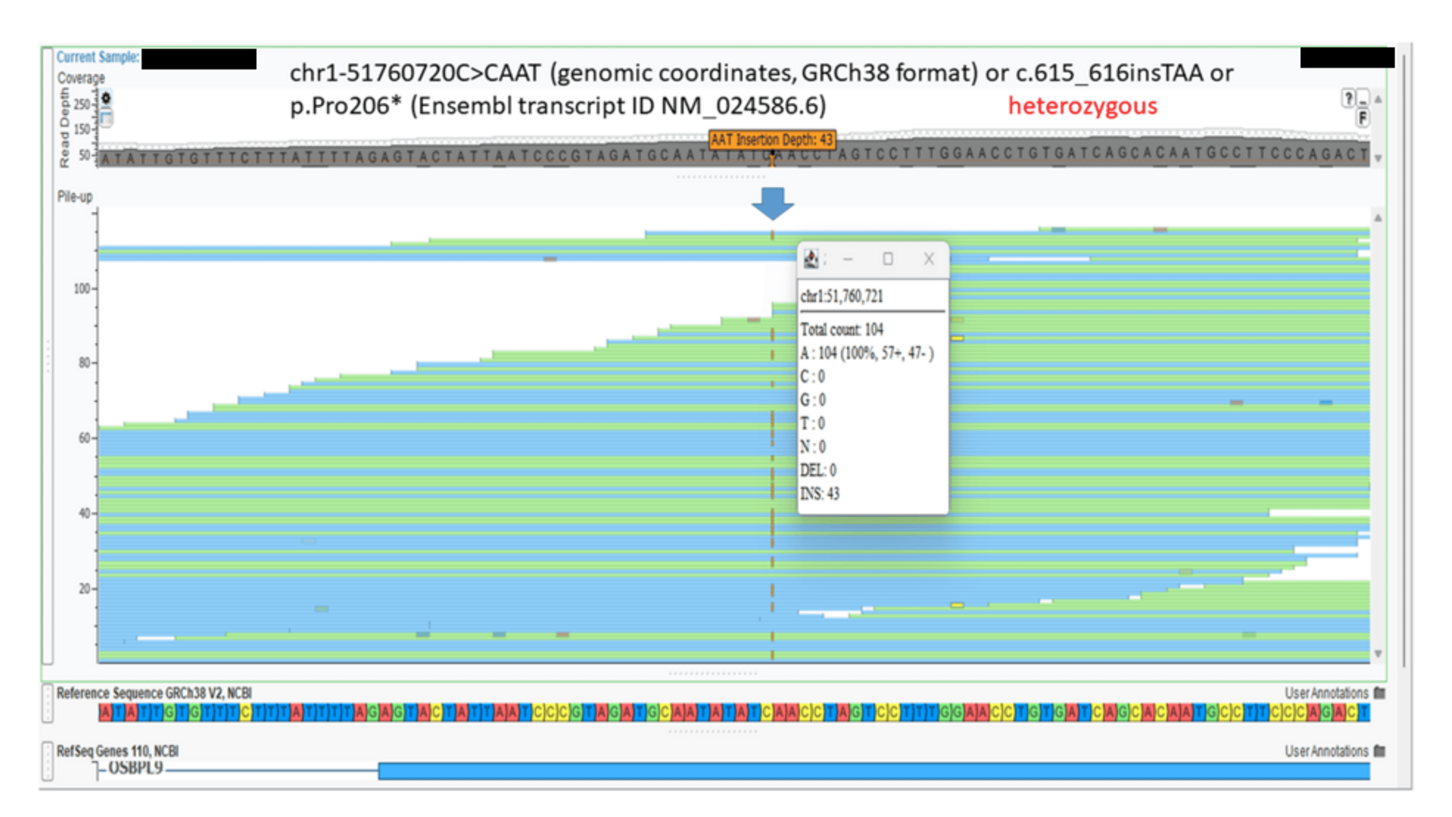
Heterozygous variant in the OSBPL9 gene identified in the father on whole exome sequencing Screenshot of the Integrated Genomic Viewer (VarSeq; Golden Helix Inc., Bozeman, Montana, US) of the binary alignment map (BAM) file of the whole exome sequence data for the carrier father shows a heterozygous mutation in the *OSBPL9* gene chr1-51760720C>CAAT (genomic coordinates, GRCh38 format) or c.615_616insTAA or p.Pro206* (Ensembl transcript ID NM_024586.6). The position of the variant is shown by the blue arrow.

This variant is absent in the gnomAD database (Appendices). No pathogenic or likely pathogenic variants for the *OSBPL9* gene have been listed in the Clinvar database. This variant was also absent in our internal database of 18000 whole exome sequencing samples. This variant is predicted to lead to the loss of function of the gene due to the absence of protein due to nonsense-mediated decay of the messenger RNA. This variant qualifies as a variant of uncertain significance classification based on the American Council of Medical Genetics classification of variants [10]. The criterion satisfied is PM2: absent or very low frequency in population databases. Whole exome analysis in the asymptomatic parents revealed them to be carriers (heterozygous) for the same variant (Figures 13, 14).

**Figure 15:**
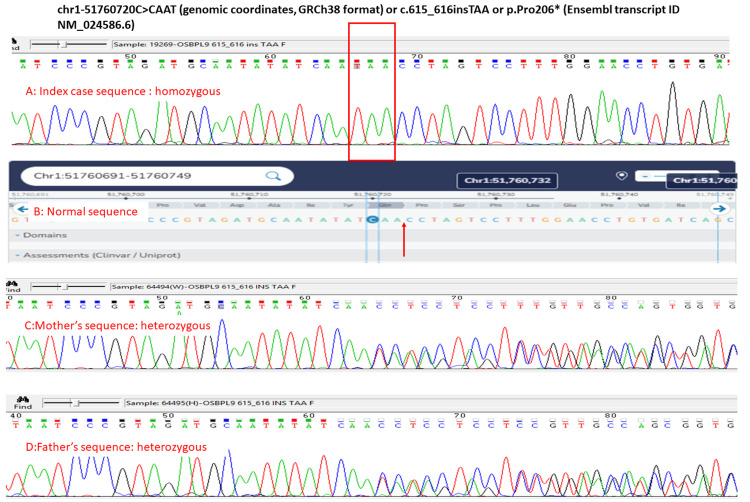
Targeted sanger sequence chromatograms for the second fetus (A: index case), normal sequence for the specific region in exon 10 of the OSBPL9 gene (B) and for the (C) mother and (D) father Sequence chromatogram for the variant in the *OSBPL9* gene: chr1-51760720C>CAAT (genomic coordinates, GRCh38 format) or c.615_616insTAA or p.Pro206* (Ensembl transcript ID NM_024586.6). In the index case (second fetus), the nucleotides TAA are inserted (homozygous variant) (highlighted by the red box) (between the nucleotides CAA on the left and CCT on the right); the position has been marked by a red arrow in the normal sequence. The parents show the same variant in the heterozygous state.

The variants were confirmed by Sanger sequencing analysis (Figure 15).

**Figure 16:**
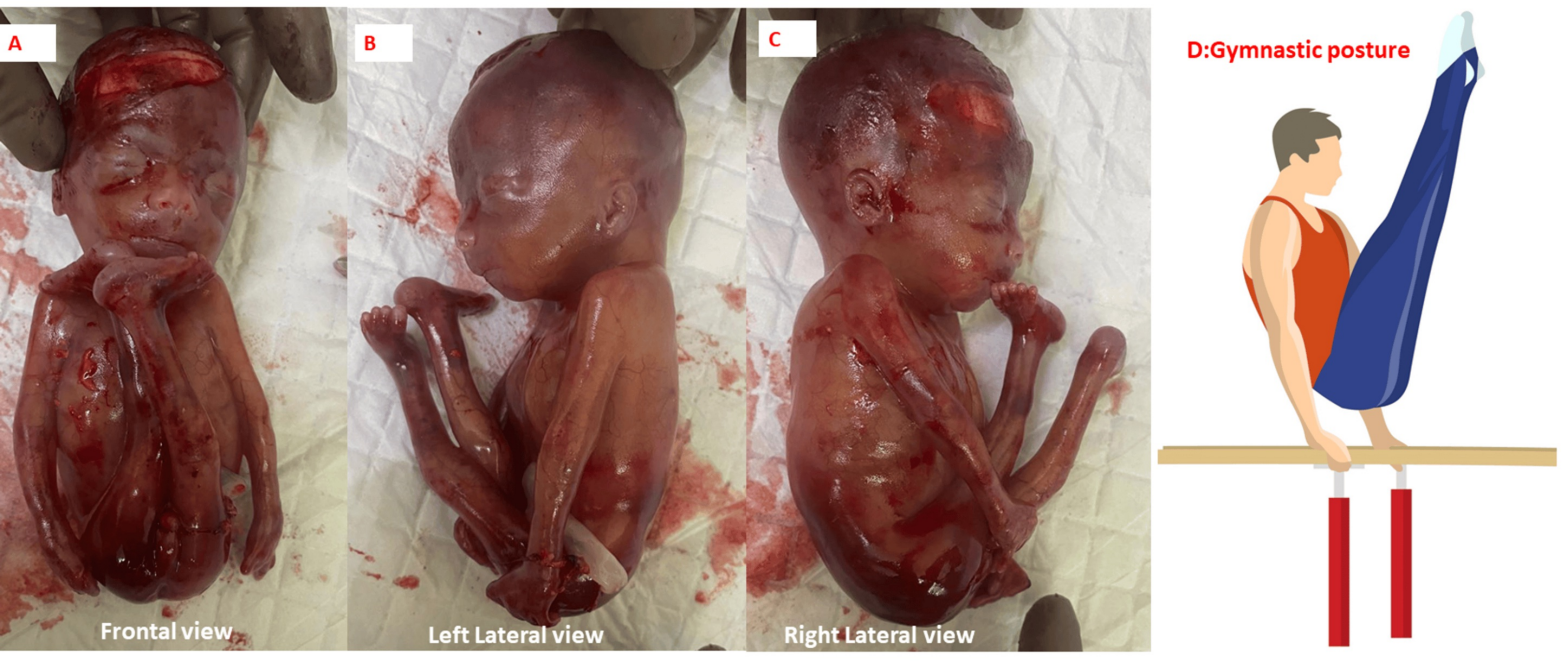
Postnatal photos of the second fetus showing facial dysmorphism, arthrogryposis multiplex in anteroposterior (A), left lateral (B), and right lateral views (C). D represents the typical posture adopted by a gymnast. Postnatal photographs of the fetus taken by the referring physician revealed a male fetus with a large head, ocular hypertelorism, long philtrum of the lip, pursed lips, arthrogryposis multiplex in the form of adducted shoulders, hyperextended elbows, clenched fists, flexed hips, hyperextended knees, and club feet bilaterally (A, B, and C). This gave the appearance of a gymnast performing on parallel bars (D). The facial photos have been presented unmasked to convey the information about facial dysmorphism and are essential to the article. Written informed consent has been obtained from the family for this and can be provided on request.

They discontinued the pregnancy as per medical advice that the fetus was likely to have adverse neurodevelopmental outcomes. Postnatal photographs of the fetus taken after delivery by the referring physician revealed a male fetus with a large head, ocular hypertelorism, long philtrum of the lip, pursed lips, arthrogryposis multiplex in the form of adducted shoulders, hyperextended elbows, clenched fists, flexed hips, hyperextended knees, and club feet bilaterally. This gave the appearance of a gymnast performing on parallel bars (Figure 16).

**Figure 17:**
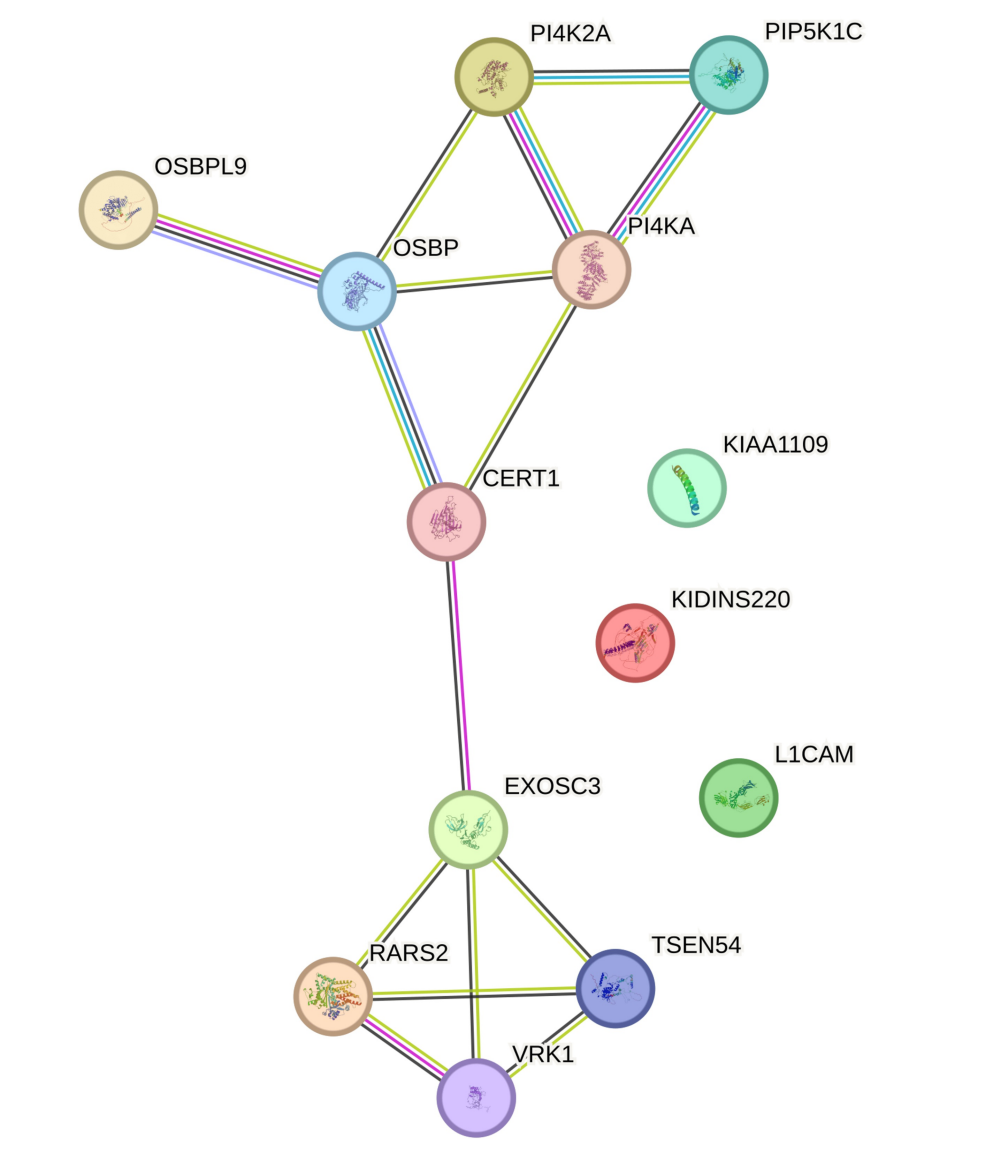
STRING (Search Tool for Retrieval of Interacting Genes/Proteins) analysis of genes known to be associated with ventriculomegaly, cerebellar hypoplasia, and arthrogryposis A STRING analysis of genes known to be associated with ventriculomegaly, cerebellar hypoplasia, and arthrogryposis was performed. The analysis found close interactions between the *OSPBPL9, OSBP, PI4K2A, PIP5K1C, PI4KA, CERT1, EXOSC3, RARS2, VRK1, *and *TSEN54* genes but no interactions with the *L1CAM, KIDINS220,* and *KIAA1109* genes. STRING: https://string-db.org

A STRING (Search Tool for Retrieval of Interacting Genes/Proteins) analysis of the genes known to be associated with ventriculomegaly, cerebellar hypoplasia, and arthrogryposis was performed. The analysis found close interactions between the *OSPBPL9, OSBP, PI4K2A, PIP5K1C, PI4KA, CERT1, EXOSC3, RARS2, VRK1,* and *TSEN54* genes but no interactions with the *L1CAM, KIDINS220*, and *KIAA1109* genes (Figure 17).

Informed consent has been obtained from the patient's family for the publication of the photos and the case information in the report. This case report was considered and granted a formal waiver by the Institutional Ethics Committee of the Dr. D.Y. Patil Medical College, Hospital and Research Centre, Dr. D.Y. Patil Vidyapeeth (Deemed to be University), Pune.

## Discussion

We thus identified a novel homozygous nonsense variant in the *OSBPL9* gene in a family with two fetuses with cerebral ventriculomegaly, cerebellar hypoplasia, and arthrogryposis multiplex inherited in a recessive manner. The segregation in the first fetus could not be confirmed due to the non-availability of stored fetal DNA. We briefly discussed the known biology of the *OSBPL9* gene. Proteins/genes showing high sequence similarity to the mammalian oxysterol binding protein (OSBP) have been identified in a variety of eukaryotic species from yeast to man. The common feature of these proteins denoted as OSBP-related proteins (ORPs) is the presence of an OSBP-type ligand binding (LB) domain. Raychaudhuri and Prinz reviewed that the ORPs are lipid transfer proteins, which include OSBP and 11 other ORPs (ORP1 to ORP11). These are implicated in many cellular processes such as signaling, vesicular trafficking, lipid metabolism, and non-vesicular sterol transport [11]. Lehto et al. (2001) used database mining to narrow down a cDNA encoding *OSBPL9*, which they called ORP9. Sequence analysis predicted that the 558-amino acid protein contains a C-terminal sterol-binding (SB) domain of around 400 residues that include the OSBP motif (Glu-Gln-Val-Ser-His-His-Pro-Pro). The OSBPL9 protein is unique and shares relatively little similarity in the SB domain with other OSBPs. The protein is highly expressed in the brain and spleen, followed by the heart, skeletal muscle, placenta, and testis [12]. Wyles and Ridgway (2004) identified two transcript variants of *OSBPL9*, namely, ORP9L and ORP9S. The ORP9L contains 723 amino acids and has a molecular mass of 81 kilodaltons, whereas ORP9S consists of 558 amino acids with a mass of 63 kilodaltons. The ORP9S is devoid of the N-terminal pleckstrin homology (PH) domain of ORP9L; however, both proteins possess a vesicle-associated membrane protein-associated protein A/B (VAP)-binding motif (also called FFAT or two phenylalanines in an acidic tract) and a C-terminal OSBP homology domain. Protein expression studies detected ORP9L in the brain, heart, kidney, and liver, and ORP9S only in the liver [13]. Ngo and Ridgway (2009) showed that the ORP9L protein is located in both the trans-Golgi network (TGN) and the endoplasmic reticulum (ER), with its specific localization within these compartments being determined by the presence of a phosphatidylinositol 4-phosphate [PtdIns(4)P]-binding PH domain, which targets it to the TGN, and a VAP-binding domain that anchors it to the ER; essentially, the protein's domains dictate where it resides within the cell. ORP9L's PH domain primarily bound PtdIns(4)P in lipid overlay assays, demonstrating ORP9L's role in PtdIns(4)P-dependent cholesterol transport between liposomes in vitro. Depletion of ORP9L via RNA interference caused Golgi fragmentation, inhibition of vesicle transport from the ER, and accumulation of cholesterol in endosomes/lysosomes. The ORP9S variant, lacking the PH domain, acted as a dominant-negative variant, causing complete protein transport cessation and cell growth inhibition upon overexpression. Ngo and Ridgway (2009) found that ORP9 maintains the early secretory pathway's integrity by facilitating sterol transport between the ER and TGN [14].

We also reviewed the OSBPL9 (mentioned as ORP9 in the paper) knockout cell knockout model by Cabukusta et al (2024) [15]. They showed that ORP9 (OSBPL9) and ORP11 form a heterodimer to localize at the ER-TGN interface and exchange phosphatidylserine (PS) for PtdIns(4)P. They showed by knocking out either the ORP9 or ORP11, de novo sphingomyelin synthesis in the Golgi apparatus diminishes whereas glucosylceramide accumulates. They found a similar effect with a knockout of the *CERT1* gene (which mediates non-vesicular trafficking of ceramide from the ER to TGN). However, it did not alter the Golgi ultrastructure as detected by light or electron microscopy. Previously, Tan and Finkel (2022) showed that ORP9 (OSBPL9) and ORP11 carry the conserved PS binding site and relocate to the lysosomal membrane to supply PS for repair during cellular stress [16]. We could not find any published study about *OSBPL9* gene knockout in mice or any other animal model. 

We briefly discussed the cases in medical literature reported with *OSBPL9* gene variants. de Ligt J et al. (2012) identified the first human patient with intellectual disability with an *OSBPL9* gene variant. The male patient was born after an uncomplicated pregnancy and delivery. Developmental delay was diagnosed at six months of age. He could sit without support at one year of age. At two years and five months, he was able to walk with support and speak five single words. He had sleeping problems. MRI of the brain was normal. He had a height of 94.5 cm (+1 SD), weight of 14 kg (-0.5 SD), and an OFC of 47.8 cm (-1.8 SD). Except for deep-set eyes and full cheeks, he did not show major facial dysmorphisms. In addition, he had fetal finger and toe pads. Microarray analysis, Angelman methylation, *UBE3A*, and *FMR1* analyses were normal. A trio exome analysis identified a de novo heterozygous variant in the *OSBPL9* gene in the child indicating a sporadic autosomal dominant phenotype (variant details: Chr1(GRCh37):g.52231512C>T, NM_148909.3, c.827C>T p.(Pro276Leu)) [17]. However, a gnomAD database search for this variant suggests that this variant is present in a heterozygous state in 71 normal individuals, indicating that this variant may not be disease-causing for an autosomal dominant phenotype [18]. However, the possibility of this variant contributing to an autosomal recessive phenotype cannot be ruled out. In another study, Monies et al (2019) identified a de novo heterozygous missense variant (NM_148906.2:c.211G>C:p.Glu71Gln) in a six years old male patient with ventricular septal defect, failure to thrive, growth retardation, ﬁne motor delay, gross motor delay, speech delay, and learning disability. This variant is absent in the gnomAD database [19]. de Aquino et al (2024) applied a genome-wide admixture mapping (AM) approach in an integrative way with rare variants (CNVs and SNVs) to extend genomic advances in autism spectrum disease (ASD) research to diverse populations. They used whole-genome sequencing data from ASD probands. They found that the predicted damaging single nucleotide variants frequency was higher in American ASD patients for *DHCR7* (0.55) and *OSBPL9* (0.21) genes, corroborating the AM findings [20].

We discussed the phenotypes of genes obtained from the STRING database analysis to show homology in the phenotype seen in our *OSBPL9* cases. Ventriculomegaly and arthrogryposis (VENARG) are caused by homozygous mutation in the *KIDINS220* gene (MIM615759) on chromosome 2p25. VENARG patients present in the antenatal period showing limb contractures consistent with arthrogryposis and enlarged brain ventricles that may be associated with hydrocephalus, abnormalities of the corpus callosum, cerebellar hypoplasia, congenital heart disease, and hydrops fetalis [21]. Neurodevelopmental disorder with hyperkinetic movements, seizures, and structural brain abnormalities (NEDMSB) is caused by a homozygous mutation in the *PI4K2A* gene (MIM609763) on chromosome 10q24. Dafsari et al. (2022) reported two unrelated girls with severe neurodevelopmental delays, failure to thrive, hypotonia, and recurrent infections in infancy. Clinical features included dysgenesis of the corpus callosum, ventriculomegaly, white matter volume loss, rotated thalami, hypoplastic anterior commissure, hypoplastic vermis, small cerebellar peduncles, hypoplastic brainstem, and mega cisterna magna [7]. Lethal congenital contracture syndrome-3 (LCCS3) is a novel autosomal recessive syndrome caused by a homozygous mutation in the *PIP5K1C* gene. It is similar to LCCS2 but lacks a distended bladder. The phenotype can be differentiated from that of LCCS1 (MIM 253310) by the absence of hydrops, fractures, and multiple pterygia. Affected individuals experience severe joint contractures, muscle wasting, atrophy, and respiratory insufficiency [22]. Neurodevelopmental disorder with spasticity, hypomyelinating leukodystrophy, and brain abnormalities (NEDSPLB) is a severe autosomal recessive disorder caused by a mutation in the *PI4KA* gene (MIM600286). Clinical features include global developmental delay, speech issues, feeding difficulties, dysmorphic facial features, seizures, and recurrent infections [23]. Neurodevelopmental disorder with hypotonia, speech delay, and dysmorphic facies (NEDHSF) is a genetic disorder caused by a heterozygous mutation in the *CERT1* gene (MIM604677). It is characterized by developmental delays, intellectual impairment, poor speech, and behavioral abnormalities. Neuroimaging frequently showed a thin corpus callosum, enlarged ventricles, delayed myelination, and cerebellar atrophy [24]. Pontocerebellar hypoplasia type 1B (PCH1B) is a severe autosomal recessive neurologic disorder causing cerebellar and spinal motor neuron degeneration at birth caused by a mutation in the *EXOSC3* gene (MIM606489). It results in diffuse muscle weakness, progressive microcephaly, global developmental delay, and brainstem involvement. Clinical features included congenital contractures, and visual impairment [25]. Pontocerebellar hypoplasia type 6 (PCH6) is a recessive genetic disorder caused by a mutation in the *RARS2* gene (MIM611524). It is characterized by delayed psychomotor development and hypotonia in the first year of life. Clinical features included seizures, and visual loss [26]. Pontocerebellar hypoplasia type 1A (PCH1A) is caused by a homozygous mutation in the *VRK1* gene (602168). Clinical features included microcephaly, developmental delay, ataxia, brisk reflexes, bilateral equinovarus, and motor and sensory neuropathy. Brain MRI showed a small cerebellar vermis and a large cisterna magna, compatible with cerebellar hypoplasia. Skeletal muscle studies showed neurogenic atrophy [27]. Mutations in the *TSEN54* gene can lead to PCH2A, PCH4, and PCH5. PCH type 2 (PCH2) is a condition characterized by progressive microcephaly, extrapyramidal dyskinesia, chorea, epilepsy, and normal spinal cord findings. A study of 88 patients with PCH2A, homozygous for the *TSEN54* p.A307S mutation, found neonatal irritability, dyskinesia, dystonia, impaired swallowing, and seizures. Pre- and perinatal complications were rare. Brain imaging showed pontocerebellar hypoplasia with a 'dragonfly-like' pattern characterized by flattened cerebellar hemispheres and a relatively preserved vermis [28]. Pontocerebellar hypoplasia type 4 (PCH4) is characterized by severe course and early lethality. Patients presented at birth with hypertonia, congenital contractures, and seizures. Brain MRI showed marked cerebellar atrophy with a peculiar cavitation in the hemispheres and vermis, and severe hypoplasia of the brainstem [29]. Patients with PCH type 5 have severe olivopontocerebellar hypoplasia and degeneration. Neuropathologic abnormalities included dysplastic, C-shaped inferior olivary nuclei, absent or immature dentate nuclei, and cell paucity more marked for the cerebellar vermis than the hemispheres. Delayed development is seen in layer 2 of the cerebral cortex and Purkinje cells of the cerebellum [30].

## Conclusions

We described a homozygous nonsense variant in the *OSBPL9 *gene in a consanguineous family with two fetuses with cerebral ventriculomegaly, cerebellar hypoplasia, and arthrogryposis multiplex, identified after chromosomal microarray (homozygosity mapping) and whole exome sequencing trio analysis. This is the first known report of a homozygous variant in the *OSBPL9* gene in a recessive inheritance pattern. Previously, two independent cases with *OSBPL9* gene variants have been documented in the literature showing a sporadic autosomal dominant pattern of inheritance. Additionally, these cases presented with intellectual disability in postnatally identified cases, and none of these were shown to have any fetal presentations such as arthrogryposis, ventriculomegaly, and cerebellar hypoplasia. This suggests that dominant and recessive variants in the *OSBPL9 *gene can have different phenotypes. Using protein-protein interaction network analysis (STRING-DB) and clinical homology with the phenotypes associated with these related genes, we suggest that the *OSBPL9* gene can be the causative gene in this case. The possible pathways in disease causation could be disturbed lipid metabolism, sphingomyelin synthesis, and lysosomal membrane repair as suggested by the role of the *OSBPL9* gene in these pathways in recent studies. The limitation of this study is the non-availability of RNA/protein/cellular/animal model studies for this variant. Further reports of *OSBPL9* gene variants in human patients will be necessary to confirm this association and deduce the exact mechanism of disease causation. Thus, this case will aid in clinical diagnosis, genetic counseling, and preventive strategies, such as prenatal diagnosis and/or preimplantation genetic diagnosis, in families identified with *OSBPL9 *gene variants.

## Figures and Tables

**Figure 18:**
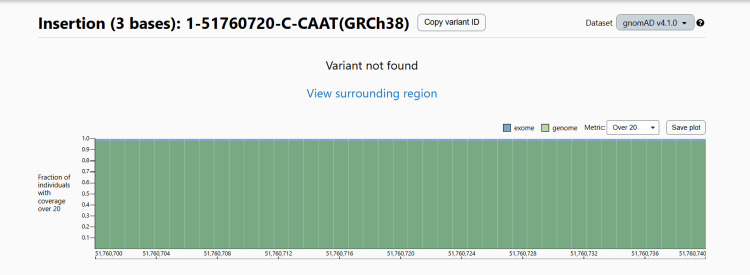
Absence of the variant in the gnomAD database Link: https://gnomad.broadinstitute.org/variant/1-51760720-C-CAAT?dataset=gnomad_r4
